# HSV-1 Encephalitis in an Elderly Man Receiving Ibrutinib for Waldenstrom's Macroglobulinemia

**DOI:** 10.1155/2020/6516037

**Published:** 2020-03-23

**Authors:** Mark R. Wallace

**Affiliations:** Skagit Valley Hospital, 360 Hospital Parkway, My Vernon, WA 98273, USA

## Abstract

Ibrutinib is a major new addition to the therapeutic armamentarium for chronic lymphocytic leukemia, mantle cell lymphoma, Waldenstrom's macroglobulinemia, and chronic graft versus host disease. Though ibrutinib has proven to be a revolutionary new small molecule agent, and has relatively minimal toxicity as compared to traditional chemotherapy, infections have emerged as a major complication of therapy. While fungal infections have been the most problematic (including CNS aspergillosis), zoster, hepatitis B reactivation, and chronic hepatitis E have been reported in association with ibrutinib therapy. This report describes a case of herpes encephalitis in an 86-year-old Waldenstrom's patient receiving ibrutinib and speculates as to whether this late life encephalitis may have been related to ibrutinib.

## 1. Introduction

Ibrutinib, an irreversible inhibitor of Bruton's tyrosine kinase (BTK), has proven to be a major advance in the treatment of multiple malignancies and graft versus host disease. Though not as toxic as standard chemotherapy, this innovative agent has, not surprisingly, been associated with a growing number of infections [1]. BTK inhibition affects not only B-cell function but also macrophage and monocyte responses, which are relevant to the control of fungal infections. Ibrutinib also irreversibly inhibits interleukin-2 inducible T-cell kinase (ITK) in CD4 cells. Absence of ITK in humans has been associated with severe herpesvirus infection [1, 2]. Invasive aspergillosis has been the most common serious infection, but other fungal pathogens (cryptococcosis, histoplasmosis, mucormycosis, and *Pneumocystis jirovecii*) have also been problematic [2–4]. Viral infections have also been an issue with the occurrence of zoster [2] as well as chronic hepatitis E and hepatitis B reactivation [5, 6]. This report details an unusual case of herpes encephalitis occurring in an 86-year-old man receiving ibrutinib for Waldenstrom's macroglobulinemia.

## 2. Case

The patient, an 86-year-old man, had a ten-year history of Waldenstrom's macroglobulinemia and type 2 diabetes. His Waldenstrom had been treated with bendamustine and rituximab, but he developed progressive anemia and eventually, pancytopenia. Ibrutinib was started seven years into his Waldenstrom, with rapid and sustained normalization of his hematocrit, peripheral white blood cells, and platelet counts. During his three years of ibrutinib therapy, he had no evidence of toxicity and led a full and active life. No other Waldenstrom therapy was provided during this period.

Three days prior to his hospital admission, the patient reported abdominal pain, anorexia, and fatigue. He was also transiently confused and on occasion had subtle difficulty with word finding. After two days of symptoms, he was evaluated at an emergency room and found to be afebrile, with normal mental status and neurologic examination. Contrast-enhanced CT scans of both his brain and abdomen were normal, and standard laboratories (complete blood count and metabolic panel) were normal. He was sent home, but the next day he developed worsening difficulty with word finding, headache, and a feeling his “mind was not working right.” An MRI of the brain revealed subtle unilateral temporal lobe enhancement, and cerebral spinal fluid had normal glucose and protein levels with a white count of 5 cells per *µ*L. Two red blood cells per *µ*L were seen; no differential was done on the white blood cells. Qualitative PCR on the spinal fluid (FilmArray Meningitis/Encephalitis panel) was positive for HSV-1. The patient was started on intravenous acyclovir at 10 mg/kg dose every 8 hours. During the period around the MRI scan and lumbar puncture he, for the first time, exhibited overt confusion and his speech became garbled. After 3 days of acyclovir, his mental status had rebounded to nearly normal, his abdominal pain resolved, and he was ready for hospital discharge. He received 21 days of acyclovir at his home. A follow-up MRI showed resolution of the temporal lobe enhancement and a repeat lumbar puncture was PCR negative for HSV-1 at the conclusion of three weeks of intravenous acyclovir therapy. There were still only 5 white cells per *μ*L, though the CSF protein was now slightly elevated at 74 mg per dL. He suffered no sequalae except for subtle memory deficits.

## 3. Discussion

This unusual case of herpes encephalitis occurred in an 86-year-old man who was receiving ibrutinib for Waldenstrom's macroglobulinemia. This case was atypical in that the patient was greater than 85 years old, had an uncommon stuttering course, and did not have a CSF pleocytosis. Lack of CSF pleocytosis occurs in 3 to 26% of patients with herpes encephalitis [7, 8].

Ibrutinib has primarily been associated with fungal infections [2, 3], but reactivation of varicella zoster, hepatitis B, and chronic hepatitis E have also emerged as issues [2, 5, 6]. A single case of West Nile encephalitis has also been reported [9] in an ibrutinib patient, suggesting that ibrutinib may predispose to severe viral infections, perhaps through irreversible inhibition of ITK. Whether this case is an isolated random occurrence, or represents the first report of herpes encephalitis as another ibrutinib related infectious risk remains to be seen. Clinicians using ibrutinib should be mindful of the risk of unusual viral as well as fungal processes and report novel infections as they occur.
